# CAR T cell therapy in solid tumors: a short review

**DOI:** 10.1007/s12254-021-00703-7

**Published:** 2021-04-08

**Authors:** Öykü Umut, Adrian Gottschlich, Stefan Endres, Sebastian Kobold

**Affiliations:** 1 Center for Integrated Protein Science Munich (CIPSM) and Division of Clinical Pharmacology, Department of Medicine IV, University Hospital, Ludwig-Maximilians-Universität München, Munich, Germany; 2 German Center for Translational Cancer Research (DKTK), partner site Munich, Munich, Germany; 3 Einheit für Klinische Pharmakologie (EKLiP), Helmholtz Zentrum München, German Research Center for Environmental Health (HMGU), Neuherberg, Germany

**Keywords:** Adoptive T cell therapy, CAR T cells, Solid tumors, Immunotherapy, Tumor immunology

## Abstract

Chimeric antigen receptor (CAR) T cell therapy has been established in the treatment of hematological malignancies. However, in solid tumors its efficacy remains limited. The aim of this article is to give an overview of the field of cell therapy itself, to introduce the underlying concepts of CAR T cell-based treatment approaches and to address its limitations in advancing the treatment for solid malignancies.

## Background

Over the last decade, treatment of cancer has undergone a radical paradigm shift. Targeted therapies, either utilizing tyrosine kinase inhibitors (TKI) or therapeutic antibodies, have developed into integral elements of oncological treatment regimes. In contrast, cellular therapies are merely starting to enter clinical routine [1]. Of these, T cell-based methods, also known as adoptive T cell therapy (ACT), are the most advanced. ACT aims to combine the extraordinary specificity of the adaptive immune system and the natural antitumor response of T cells in the fight against cancer. To date, depending on the source of the T cells and the subsequent genetic or nongenetic manipulation, three main forms of T cell-based therapies can be distinguished:

- Tumor-infiltrating lymphocytes (TIL),
- T cell receptor (TCR)-engineered T cells and
- Chimeric-antigen receptor (CAR) T cells [2].

TIL are T cells, found in the tumor tissue, which in most cases are equipped with endogenous TCR specific for tumor-associated antigens. In TIL-based ACT, these T cells are isolated from surgical tumor specimens, are expanded in vitro and re-infused into the patients [3]. However, one major limitation to this approach is the often low number of antigen-specific T cells found in tumor explants and the inability to retrieve and expand T cells from all patients. To overcome these limitations, in vitro engineering methods have been developed to create antigen-specific T cells without needing to isolate them from tumor tissues. As such, naïve, unspecific T cells are isolated from the peripheral blood of the patients via leukapheresis, are then genetically modified with a tumor-specific recognition construct (e.g., tumor-specific TCR, CAR), expanded and finally re-infused into the patient [4].

## TCR-engineered T cells and CAR T cells

As described, TCR T cells are genetically modified to express an antigen-specific TCR. In the treatment of neoplastic diseases, the target is usually a tumor-specific antigen (TSA) or tumor-associated antigen (TAA). Ideally, these would be uniquely expressed in malignant cancer cells, but not in healthy cells. Peptides derived from TSA are generated through intracellular proteasome-mediated processing mechanisms and subsequently presented on the MHC‑I complex of the tumor cells [2]. TCR-engineered T cells are able to recognize the MHC-TSA-peptide complex, which leads to an activation of T cells and subsequent lysis of neoplastic cells. In contrast, CAR T cells are engineered through introduction of an artificial synthetic construct, which ultimately also leads to the activation of the T cells and tumor cell lysis [2]. Both strategies have certain advantages and disadvantages, which have been extensively reviewed elsewhere [2, 5].

**Fig. 1:**
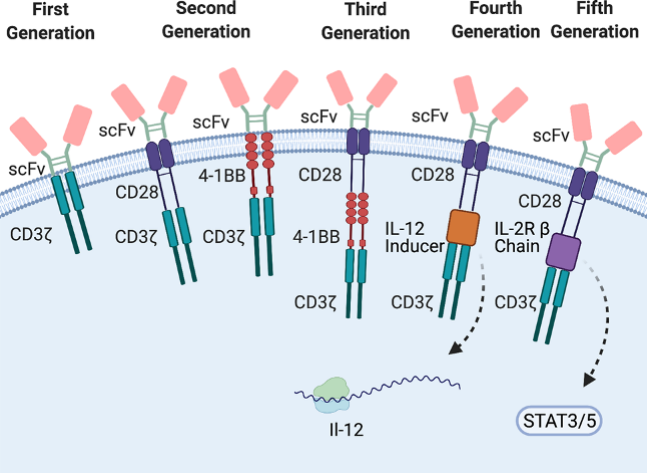
Structure and classification of CAR T cells. CAR T cells are grouped into different generations depending on the structure of the CAR. Recent advancements have added new CAR structures, which are extensively reviewed in [5, 8]. *scFv* single chain variable fragment, *CD3ζ* CD3 zeta chain, *IL-2R β* *Chain* IL‑2 receptor β chain

The artificial CAR construct usually contains an antibody-derived single-chain variable fragment (scFv) as an extracellular domain, a hinge domain and a transmembrane domain, anchoring the receptor in the cell membrane. T cells expressing the construct are able to bind the respective TSA via the extracellular antibody-derived scFv domain of the CAR receptor. Activation of T cells is subsequently induced by an intracellularly located signaling domain, consisting of a CD3ζ chain and one or more co-stimulatory domains (e.g., CD28, 4‑1BB) [2]. The CD3ζ chain is physiologically part of the TCR-CD3 complex and is the major inducer of T cell activation following antigen recognition. Co-stimulatory domains were included in the second and third generations of CAR constructs, as augmented antitumor efficacy [6] and increased persistence of the transferred T cells [7] has been observed. Depending on the CAR receptor used, CAR T cells are classified in different generations, as depicted in Fig. 1.

In recent years, further improvements of the CAR structures have been employed in order to improve efficacy of CAR T cells, especially in solid malignancies (see “CAR T cells in solid tumors” section). These innovative approaches have been extensively reviewed by us and other groups and interested readers are referred to the following literature for a more detailed overview [5, 8]. In short, fourth-generation CAR constructs incorporate a cassette into the intracellular domain to induce the secretion of pro-inflammatory cytokines. This strategy enables the innate immune system to contribute to the antitumor effect (Table 1; [9]). In contrast, fifth-generation CAR T cells followed a completely different approach and aimed to activate the Janus kinase-signal transducers and activators of transcription (JAK-STAT) signaling pathway to promote T cell proliferation. Fifth-generation CAR T cells were shown to have superior antitumor effect and persistence compared to second- and third-generations [10]. However, these developments are merely at the beginning and not part of the clinical routine. All U.S. Food & Drug Administration (FDA)- or European Medicines Agency (EMA)-approved ACT strategies are second-generation CAR-based approaches and only approved for the treatment of certain hematological malignancies.

**Table 1:** Published clinical trials in solid tumors

Trial number	Cancer entity	Published	CAR Target molecule	Trial Phase	Patients *N*	Outcome	Reference
NCT01869166	Biliary tract cancer	2018	EGFR	I	19	1/17 complete remission 10/17 stable disease	[36]
NCT02349724	CRC	2017	CEA	I	10	7/10 stable disease	[15]
NCT01212887	GI tumors	2017	CEACAM5	I	14	No objective clinical response Terminated due to safety concerns	[37]
NCT02541370	GI tumors	2018	CD133	I	23	3/23 partial response 14/23 stable disease	[38]
NCT00730613	Glioblastoma	2015	IL13Rα2	I	3	No objective clinical response	[16]
NCT02209376	Glioblastoma	2017	EGFRvIII	I	10	Not available due to surgical intervention	[17]
NCT01109095	Glioblastoma	2017	HER-2/neu	I	17	1/17 partial response 7/17 stable disease	[39]
NCT01454596	Glioblastoma	2019	EGFRvIII	I	18	No objective clinical response	[21]
NCT02395250, NCT03146234	HCC	2020	GPC3	I	13	2/13 partial response, 1/13 stable disease	[40]
Park et al.	Neuroblastoma	2007	L1-CAM	I	6	1/6 stable disease then partial response	[41]
Pule et al.	Neuroblastoma	2008	GD2	I	11	4/8 evidence of regression	[42]
NCT00085930	Neuroblastoma	2011	GD2	I	19	3/19 complete remission	[43]
NCT01822652	Neuroblastoma	2017	GD2	I	11	5/11 stable disease	[44]
NCT01869166	NSCLC	2016	EGFR	I	11	2/11 partial response 5/11 stable disease	[18]
Kershaw et al.	Ovarian Carcinoma	2006	FRα	I	14	No objective clinical response	[45]
NCT01897415	PDAC	2018	MSLN	I	6	2/6 stable disease	[46]
NCT01869166	PDAC	2020	EGFR	I	14	4/14 partial response 8/14 stable disease	[14]
Junghans et al.	Prostate cancer	2016	PSMA	I	5 (6)	2/5 partial response	[47]
Lamers et al*.*	RCC	2016	CAIX	I	12	No objective clinical response	[48]
NCT00902044	Sarcomas	2015	HER-2/neu	I	17 (19)	4/17 stable disease	[49]
NCT02159716	Solid tumors	2019	MSLN	I	15	11/15 stable disease	[50]

*CRC* Colorectal Carcinoma, *GI tumor*, Gastrointestinal Tumor, *HCC* Hepatocellular Carcinoma, *NSCLC* Non-Small Cell Lung Cancer, *PDAC* Pancreatic Ductal Adenocarcinoma, *RCC* Renal Cell Carcinoma, *IL13Ra2* Interleukin-13 receptor subunit alpha 2, *L1-CAM* L1 Cell Adhesion Molecule, *HER2/neu* Human epidermal growth factor receptor 2, *EGFR(vIII)* Epidermal Growth Factor Receptor (variant III), *CEACAM5* Carcinoembryonic antigen-related cell adhesion molecule 5, *MSLN* Mesothelin, *CEA* Carcino-Embryonic Antigen, *GPC3* Glypican‑3, *CAIX* Carboxyanhydrase-IX, *FRα* α-folate Receptor, *PSMA* Prostate-specific membrane antigen

## CAR T cells in hematological malignancies

Axicabtagene ciloleucel and Tisagenlecleucel, both approved in 2017 by the FDA and in 2018 by the EMA, are CAR T cells engineered to target the B cell lineage antigen CD19. CD19 is exclusively expressed on both healthy and malignant B cells. Consequently, these CAR T cells can be used to treat B cell malignancies such as diffuse large B cell lymphomas (DLBCL) and B cell acute lymphoblastic leukemia (B-ALL, only Tisagenlecleucel). Approval was granted after astonishing initial response rates of up to 93% in ALL and 54% in DLBCL were observed [5]. Importantly, these response rates were reached in extensively pretreated patients with chemotherapy-refractory or relapsed malignant disease and many were durable [2]. A third T cell product was just recently approved by both the FDA and the EMA for the treatment of mantle cell lymphoma (MCL; Tecartus, brexucabtagene autoleucel) (NCT02601313) [11]. Recent long-term follow-up studies revealed sustained response rates in patients. However, disease relapse is seen in up to 41% of patients suffering from ALL [12]. In contrast, response rates in DLBCL seems to be more durable as the majority of responding patients do not experience relapse during the 12-month follow-up period [5, 13]. In summary, CAR T cell therapy has emerged as an important therapeutic option for hematological malignancies. However, in nonhematological malignancies CAR T cell therapy has so far failed to demonstrate comparable treatment responses.

## CAR T cells in solid tumors

Encouraged by the striking results seen in DLBCL and ALL, new CAR T cells targeting different epithelial antigens were developed and clinically tested. As such, CAR-based ACT was evaluated in different gastrointestinal malignancies (pancreatic cancer, NCT01869166; colorectal cancer, NCT02349724) [14, 15], glioblastoma (NCT00730613; NCT02209376) [16, 17] and non-small cell lung cancer (NSCLC, NCT01869166) [18]. Table 1 gives a summary of already conducted clinical trials in solid malignancies.

Treatment and outcomes of patients suffering from carcinomas, per definition derived from epithelial tissues, however, differs from the treatment of hematological malignancies. To at least some extent, target antigens are usually co-expressed on healthy tissues [5]. Application of anti-CD19 CAR T cells for example can lead to sustained B cell aplasia. In the clinical setting however, this is not a life-threatening side effect and can be managed with regular substitution of immunoglobulins [19]. In comparison, in solid malignancies the target antigen can be expressed at low levels on other epithelial cells (e.g., lung, heart) and thus potentially lead to serious adverse effects, as observed by Morgan et al. [20]. Furthermore, high-dose treatment with CAR T cells against an epithelial cell antigen (EGFRvIII) has also been reported to lead to the congestion of pulmonary vasculature and lethal respiratory failure (NCT01454596) [21].

As a consequence, several newly initiated clinical trials have primarily focused on the safety of the newly developed CAR T cells, directed against various target antigens of solid tumors (e.g., EGFRvIII, MUC‑1, MAGE, CEA, GD2, CA125, MSLN; Table 1; [22]). These studies were most often conducted in malignancies with poor overall survival such as glioblastoma and pancreatic ductal adenocarcinoma; however, as depicted in Table 1, therapies are assessed in a wide range of different solid malignancies. While most treatments were shown to be safe, the overall response rates observed in these trials, especially compared to the impressive clinical benefit obtained in ALL and DLBCL, were rather disappointing. Overall mortality remained approximately the same and the patients usually only benefited from the treatment temporarily [8].

## Hurdles of CAR T cell therapy in solid tumors

As described above the responsiveness of solid malignancies to CAR T cell therapy is bleak at best.

Over the last years, researchers have identified several underlying mechanisms responsible for the lack of treatment efficacy in solid tumors and have identified three major hurdles: (1) trafficking of T cells as the first key limiting step, (2) the choice of target antigen and antigen loss (tumor cell recognition) and (3) the hostile tumor microenvironment [8].

Trafficking of the transferred T cells into solid tumors is a limiting factor dampening therapeutic efficacy. As such, different strategies have been applied to improve T cell trafficking into the tumors. Direct application (intratumoral injection) of T cells has been employed to directly deliver the CAR T cells to the tumor site (NCT00730613) [16]. The need for invasive interventional procedures as well as the often inaccessible tumors however limit these approaches. Alternative strategies make use of physiological processes of immune cell trafficking: Immune cell recruitment to the site of inflammation is mediated by the chemokine–chemokine receptor axis. High levels of chemokine ligands secreted at the site of inflammation lead to the recruitment of immune cells expressing matching receptors. As solid tumors tend to show enhanced levels of chemokine ligands, co-transduction of chemokine receptors commonly not present on T cells, and CAR receptors into T‑cells, has been employed by us and another groups [23, 24]. This has been shown to enhance both T cell infiltration and therapeutic efficacy in preclinical models. This concept is currently under investigation in clinical trials (NCT03602157).

Loss of the target antigen on tumor cells is a problem common in treatment of both hematological and nonhematological malignancies. Relapse with CD19-negative disease, for example, is frequently observed after treatment with CD19 CAR T cells. In solid tumors, down-regulation of the target antigen following CAR T cell therapy has also been reported in different clinical trials [17]. Targeting of multiple antigens (e.g. CD19 plus CD20; CD19 plus CD22) or alternatively sequential targeting strategies have shown benefit in different clinical trials [25–27].

Finally, solid tumors exhibit a complex, often hostile tumor microenvironment (TME). Besides cancer cells, the TME of solid tumors comprises infiltrating and resident immune cells, stromal cells as well as many pro- and anti-inflammatory mediators [28]. The interactions between the different components of the TME are complex and cannot be described in detail here. For further information please see the following literature [28–30]. In general, the components of the TME suppress an appropriate immune response against cancer cells, thus, creating a conducive environment for the tumor cells to proliferate.

The nowadays commonly used checkpoint inhibitors (e.g., pembrolizumab, nivolumab, ipilimumab) boost the activation and function of T cells through blockade of inhibitory receptors on T cells (e.g., PD‑1, CTLA-4). As such, combining CAR T cells with immune checkpoint inhibitors or other drugs influencing the immunosuppressive nature of the TME are currently being investigated [8]. In addition, genetic engineering can be employed to lift immune-suppressive effects on the transferred T cells. Our group has developed a fusion receptor, switching the inhibitory signal of PD‑1 into a T cell activating signal [31]. Alternatively, CRISPR-Cas9-mediated disruption of the PD‑1 locus in CAR T cells has been shown to increase therapeutic efficacy of CAR T cells in vitro and in vivo. Several clinical trials are currently investigating these strategies (NCT03081715, NCT02867332, NCT02867345, NCT02793856, NCT03044743) [32].

Lastly, preclinical research has demonstrated the feasibility of redirecting CAR T cells not against tumor cells, but at immunosuppressive cells in the tumor microenvironment. Thus, CAR T cells targeting cancer-associated fibroblasts or tumor-associated macrophages have been shown to delay cancer progression in preclinical mouse models [33, 34]. However, to date no clinical data on these strategies are available, so the value remains uncertain.

## Conclusion

The clinical transition of CAR T cell therapy has started a new era in oncology. Although these approaches have already given hope to incurable cancer patients suffering from hematological malignancies, it still remains to be proven in the comprehensive field of solid malignancies. As one might infer from our short overview, glioblastoma was often targeted in clinical studies. Due to limitations arising from its anatomical location and quick progression rate, glioblastoma remains clinically challenging to this date. Even a tumor as aggressive as glioblastoma was reported to be fully regressed in a case collection by Brown et al. [35]. The overall results of clinical studies might seem disappointing, but such case reports highlight the potential of CAR T cell therapy in solid cancers and maybe give a glimpse into what can be achieved in the future. Consequent advancement of promising preclinical strategies into clinical testing is now crucial to broaden the scope of cellular therapies and to increase the efficacy in solid tumors, with the hope that these therapies will not only be effective in single patients, but present a real clinical alternative for so many incurable cancer patients in daily oncological routine.

### Take home message

- Adoptive T cell therapy has emerged has an important treatment option in relapsed and chemotherapy-refractory hematological malignancies.
- Clinical trials in solid tumors have primarily focused on establishing the safety of CAR T cell therapy; however, secondary endpoint analyses have so far only revealed modest efficacy.
- Preclinical research has been able to identify major caveats of CAR T cell therapy in solid tumors. Clinical trials will now have to determine whether this can be translated into clinically relevant improvements in patient outcome.
